# Supplementary material for: Differential adhesion regulates neurite placement via a retrograde zippering mechanism
Source: eLife. 2021 Nov 16;10:e71171. doi: 10.7554/eLife.71171 (PMC8843091; doi:10.7554/eLife.71171)
Supplement: Supplementary file 1. [file elife-71171-supp1.docx]

Supplementary File 1

| Plasmid name | Full construct | Source |
| --- | --- | --- |
| DACR1412 | Pinx-1(0.5 kb)::mCherry::unc-54UTR | ([Moyle et al., 2020](#_ENREF_56)) |
| DACR2245 | Pinx-1(1 kb)::eGFP::rab-3::unc-54UTR | ([Moyle et al., 2020](#_ENREF_56)) |
| DACR2796 | Pinx-1(451 bp)::glr-1::GFP::unc-54UTR | This paper |
| DACR2651 | Pinx-1(1 kb)::mCh::rab-3::unc-54UTR | This paper |
| DACR199 | Pcex-1::GFP | This paper |
| DACR3149 | Pcex-1::mTagBFP1::unc-54UTR | This paper |
| DACR3529 | Psyg-1::PHD::GFP::unc-54UTR | This paper |
| DACR3492 | Pinx-19::p12-caspase3::unc-54UTR | This paper |
| DACR3493 | Pinx-19::p17-caspase3::unc-54UTR | This paper |
| DACR3505 | Ptdc-1::p17-caspase3::unc-54UTR | This paper |
| DACR2637 | Ptdc-1::GFP::unc-54UTR | This paper |
| DACR2863 | Ptdc-1::PHD::GFP::unc-54UTR | This paper |
| DACR2704 | Pinx-19::PHD::GFP::unc-54UTR | This paper |
| DACR2481 | Pnphp-4::PHD::GFP::unc-54UTR | This paper |
| DACR2404 | Pinx-1(1 kb)::mCh::PHD::unc-54UTR | This paper |
| DACR2371 | Punc-42::PHD::GFP::unc-54UTR | This paper |
| DACR2607 | Punc-42::ZF1::PHD::GFP::unc-54UTR | ([Moyle et al., 2020](#_ENREF_56)) |
| DACR2609 | Plim-4(includes exons 1-3)::SL2::Zif1::unc-54UTR | ([Moyle et al., 2020](#_ENREF_56)) |
| DACR2351 | Plim-4(includes exons 1-3)::mCherry::unc-54UTR | ([Moyle et al., 2020](#_ENREF_56)) |
| DACR3605 | Ptdc-1::mScarlet::PHD::unc-54UTR | This paper |
| DACR3502 | Pmgl-1b::syg-1b::unc-54UTR | This paper |
| DACR3503 | Pnphp-4::syg-1b::unc-54UTR | This paper |
| DACR3698 | Pmgl-1b::syg1ecto::unc-54UTR | This paper |
| DACR2618 | Pinx-1(451 bp)::cla-1::GFP::unc-54UTR | This paper |
| DACR3527 | Pinx-1(1 kb)::syg-1b::unc-54UTR | This paper |
| DACR3780 | Psyg-1::syg-1::GFP::unc-54UTR | This paper |
| DACR3781 | Pmgl-1b::syg-1endo::unc-54UTR | This paper |
| DACR3888 | Pinx-1(1 kb)::syg-2b::unc-54UTR | This paper |
| DACR3890 | Pinx-1(1 kb)::GFP::syd-2::unc-54UTR | This paper |
| DACR3901 | Pinx-1(1 kb)::cla-1::GFP::unc-54UTR | This paper |
