# Supplementary material for: Differential adhesion regulates neurite placement via a retrograde zippering mechanism
Source: eLife. 2021 Nov 16;10:e71171. doi: 10.7554/eLife.71171 (PMC8843091; doi:10.7554/eLife.71171)
Supplement: Transparent reporting form [file elife-71171-transrepform1.pdf]

Westbrook Centre
Milton Road
Cambridge CB4 1YG, UK

P 01223 855340
W elifesciences.org
T @elife

eLife's transparent reporting form

We encourage authors to provide detailed information *within their submission* to facilitate the interpretation and replication of experiments. Authors can upload supporting documentation to indicate the use of appropriate reporting guidelines for health-related research (see EQUATOR Network), life science research (see the BioSharing Information Resource), or the ARRIVE guidelines for reporting work involving animal research. Where applicable, authors should refer to any relevant reporting standards documents in this form.

If you have any questions, please consult our Journal Policies and/or contact us: editorial@elifesciences.org.

Sample-size estimation

- You should state whether an appropriate sample size was computed when the study was being designed
- You should state the statistical method of sample size computation and any required assumptions
- If no explicit power analysis was used, you should describe how you decided what sample (replicate) size (number) to use

Please outline where this information can be found within the submission (e.g., sections or figure legends), or explain why this information doesn't apply to your submission:

The phenotypes examined have large effect sizes, as determined by computing the Cohen's d statistic and the sample sizes used captured significant differences between phenotypes. Scoring was done blind to genotype, and by multiple independent researchers to confirm and compare observations. Scoring was done across different days, and by examining/noting developmental stages, to control for variables that might affect the robust phenotypes observed. This information can be found in the Methods section.

Replicates

- You should report how often each experiment was performed
- You should include a definition of biological versus technical replication
- The data obtained should be provided and sufficient information should be provided to indicate the number of independent biological and/or technical replicates
- If you encountered any outliers, you should describe how these were handled
- Criteria for exclusion/inclusion of data should be clearly stated
- High-throughput sequence data should be uploaded before submission, with a private link for reviewers provided (these are available from both GEO and ArrayExpress)

Please outline where this information can be found within the submission (e.g., sections or figure legends), or explain why this information doesn't apply to your submission:

In this study we examine single embryos or larvae, each representing independent biological replicates. The number of animals (biological replicates) examined are indicated in the figure legends. All data points were reported for each experiment and no outliers were excluded from the reports. All transgenic strain markers created and used for this study were vetted by comparing the phenotypes in wild type animals with known electron microscopy connectomic studies and other controls. Cellular and subcellular markers were characterized in multiple transgenic lines of the same strain. In creating extrachromosomal transgenic lines, expression levels of markers displayed mosaic patterns, preventing quantifications of desired phenotypes in one neuron of the AIB neuron pair for 1, 2 and 4 WT animals for the analyses in Figure 4-figure supplement 1B, Figure 3C, and Figure 7J. Additionally, during imaging of *syg-2(ky671)* animals for the analysis in Figure 4M, 5 animals moved between stacks, preventing quantifications of neurite positions, and were therefore excluded. All experiments were performed over several days, looking at multiple generations of the same strains for consensus/robustness/reproducibility of the observed phenotypes.

Statistical reporting

- Statistical analysis methods should be described and justified
- Raw data should be presented in figures whenever informative to do so (typically when N per group is less than 10)
- For each experiment, you should identify the statistical tests used, exact values of N, definitions of center, methods of multiple test correction, and dispersion and precision measures (e.g., mean, median, SD, SEM, confidence intervals; and, for the major substantive results, a measure of effect size (e.g., Pearson's r, Cohen's d)
- Report exact p-values wherever possible alongside the summary statistics and 95% confidence intervals. These should be reported for all key questions and not only when the p-value is less than 0.05.

Please outline where this information can be found within the submission (e.g., sections or figure legends), or explain why this information doesn’t apply to your submission:

For all experiments, statistical tests used, exact values of N, exact p-values and methods of multiple test correction are reported in Figure legends. Dispersion and precision measures used in plots have also been specified in Figure legends. Effect size was measured using Cohen’s d statistic and indicated in Figure legends.

(For large datasets, or papers with a very large number of statistical tests, you may upload a single table filewith tests, Ns, etc., with reference to sections in the manuscript.)

Group allocation

- Indicate how samples were allocated into experimental groups (in the case of clinical studies, please specify allocation to treatment method); if randomization was used, please also state if restricted randomization was applied

eLife Sciences Publications, Ltd is a limited liability non-profit non-stock corporation incorporated in the State of Delaware, USA, with company number 5030732, and is registered in the UK with company number FC030576 and branch number BR015634 at the address Westbrook Centre, Milton Road, Cambridge CB4 1YG.

- Indicate if masking was used during group allocation, data collection and/or data analysis

Please outline where this information can be found within the submission (e.g., sections or figure legends), or explain why this information doesn't apply to your submission:

C. elegans were divided by the indicated genotypes (which were all confirmed by genotyping), or by the developmental stage of the animal as determined by minutes post fertilization for embryos (described in Duncan et al., 2019) and stereotyped standard anatomical features for larval stages, as indicated in the Methods section.

Additional data files ("source data")

- We encourage you to upload relevant additional data files, such as numerical data that are represented as a graph in a figure, or as a summary table
- Where provided, these should be in the most useful format, and they can be uploaded as "Source data" files linked to a main figure or table
- Include model definition files including the full list of parameters used
- Include code used for data analysis (e.g., R, MatLab)
- Avoid stating that data files are "available upon request"

Please indicate the figures or tables for which source data files have been provided:

Source data has been provided for all plots in which individual data points are not represented – Figure 4N, Figure 6K, Figure 1-figure supplement 2I, Figure 1-figure supplement 2J, Figure 5-figure supplement 4N, Figure 6-figure supplement 1C, Figure 7-figure supplement 3N

eLife Sciences Publications, Ltd is a limited liability non-profit non-stock corporation incorporated in the State of Delaware, USA, with company number 5030732, and is registered in the UK with company number FC030576 and branch number BR015634 at the address Westbrook Centre, Milton Road, Cambridge CB4 1YG.
